# Unmet Supportive Care Needs in Cancer Survivors in Spain: A Multicentre Cross-Sectional Study on Prevalence and Sociodemographic and Disease-Related Risk Factors

**DOI:** 10.3390/curroncol32090524

**Published:** 2025-09-19

**Authors:** Yolanda Andreu, Beatriz Gil-Juliá, Carmen Picazo, Ana García-Conde, Ana Soto-Rubio

**Affiliations:** 1 Department of Personality, Assessment and Psychological Treatments, Faculty of Psychology and Speech Therapy, University of Valencia, 46010 Valencia, Spain; yolanda.andreu@uv.es (Y.A.); beatriz.gil@uv.es (B.G.-J.); 2 Department of Psychology and Sociology, Faculty of Social and Human Sciences of Teruel, University of Zaragoza, 44003 Teruel, Spain; carmen.picazo@unizar.es; 3 Fundación Instituto Valenciano de Oncología, 46009 Valencia, Spain; ana.garcia-conde@uv.es; 4 Department of Developmental and Education Psychology, Faculty of Psychology and Speech Therapy, University of Valencia, 46020 Valencia, Spain

**Keywords:** cancer survivors, quality of life, unmet care needs, supportive care, risk factors

## Abstract

Objective: This multicentre study investigates unmet supportive care needs (SCNs) among cancer survivors in Spain and analyses sociodemographic and cancer-related risk factors. Methods: A cross-sectional design was used with 1862 cancer survivors aged 18–92 years who had completed primary treatment with curative intent and were disease-free. Participants responded to the Cancer Survivors’ Unmet Needs (CaSUN) questionnaire. Descriptive and multivariate analyses explored SCNs in the total sample and subgroups, as well as differences according to sociodemographic and cancer-related variables. Results: At least 20% of participants reported 18 needs out of a total of 35 identified by the CaSUN questionnaire. One-third to half reported needs in the comprehensive care and information domain. Risk factors for reporting more needs included younger age; female sex; not having a partner; being on sick leave or unemployed; having a diagnosis of haematological, breast or gynaecological cancer; receiving systemic treatment (chemotherapy and/or hormone therapy); and being at an earlier stage of survival. Conclusions: The study highlights significant unmet care needs among cancer survivors in Spain and the urgency of improving management of the physical and psychosocial effects of cancer and its treatment. Special attention should be given to those at greatest risk through personalised and comprehensive care strategies integrated into survivorship programs.

## Simple Summary

People who have successfully completed hospital treatment for cancer face multiple challenges that may still be present even several years later. These include fear of disease recurrence, the physical effects of treatment—pain, fatigue and cognitive impairment—and, consequently, difficulties in resuming family, social and work roles. Thus, care in cancer survivorship should be comprehensive and address those care needs, physical and psychosocial, that cancer survivors perceive as unmet, as well as determine their possible risk factors for their presence that allow prioritising attention in the most vulnerable subgroups. Studies at the national level are of special relevance since the experience with cancer is influenced by factors such as culture or the existing healthcare system. Therefore, this paper explores unmet care needs and their possible risk factors in cancer survivors in Spain, with the aim that the information obtained may guide future health policies in this country.

## 1. Introduction

The steady increase in worldwide cancer survival rates [1,2] has prompted growing interest in understanding and improving health-related quality of life (HRQOL) during the cancer survival period [3,4]. People who complete primary cancer treatment, even if it has been successful, face a variety of adverse side effects that affect their physical, psychological and social functioning, resulting in unmet supportive care needs (SCNs) during their survival period [5,6,7]. Accordingly, the challenge of achieving the highest possible levels of care to improve HRQOL in cancer survival involves (i) determining the SCNs existing in this period as well as (ii) identifying possible variables that identify risk subgroups on which to prioritise care.

Overall, the results so far show that one of the most common SCNs detected is related to fear of cancer recurrence [8,9,10,11,12,13]. In addition, other particularly prominent SCNs are linked to insufficient information about the disease, treatment and relapse symptoms that is necessary to resume life [8,12,14,15,16], as well as psychological support for managing the disease experience [17], comprehensive and coordinated medical care [11,18,19], assistance related to physical changes experienced that make it difficult to carry out usual activities [12,20,21,22,23,24] and financial support [25,26].

In terms of sociodemographic and disease-related risk factors, although the results are not always consistent, most studies point to a greater presence of SCNs in younger individuals [7,11,24,27,28,29,30,31,32,33,34,35], women [7,31,36], those who are unmarried or without a partner [27,37,38], and those who are unemployed [39,40]. Some studies report more frequent SCNs in survivors of certain types of cancer, such as breast [25,33,40], head and neck [13], gynaecological, and colorectal cancer [41], as well as in those survivors whose treatment includes systemic treatments such as chemotherapy [11,40,42] or adjuvant hormone therapy (AHT) [43]. Finally, the results regarding post-treatment time are mixed. While some studies have found an association between shorter elapsed time and reporting more unmet needs [31,44], others have found no such association [16,25].

Despite the above results, some weaknesses in existing studies, two of which stand out, limit the ability to establish conclusions regarding the prevalence of SCNs and their associated factors [33]. A first relevant issue is that the lack of consistency in the measurement, classification and reporting of unmet needs makes comparisons difficult [16,44,45,46]. The examination of SCNs is particularly complex because, in addition to quantitative analysis, it also involves a relevant qualitative analysis to determine the specific needs that require the most attention. It is for this reason that formal assessment is crucial in identifying and addressing SCNs [37,47,48]. Currently available tools are not fully satisfactory in terms of content and structural validity, internal consistency, and the conceptual framework that informed their development [42]. Some studies use non-validated scales [42] or non-validated translated versions [11]. Other studies report the frequent use of self-constructed questionnaires, with great heterogeneity in categories, development and quality [45]. The use of psychometrically satisfactory instruments and the exploration of the prevalence of SCNs both (i) by content areas, thus outlining the most deficient areas, and (ii) by specific needs that make explicit those particular issues in which the demand for help is greater are of special interest to facilitate the comparison of results and the advancement of research.

A second, even more relevant issue, which hinders the comparability of the results obtained, is the lack of a universally accepted definition of “cancer survivor” [44,49]. This conceptual imprecision adds an additional source of variability. For example, the definition most closely linked to the medical tradition understands a survivor to be a person who has remained free of the disease for more than 5 years [50], thus not including those persons who, having overcome the disease, have not reached this time limit. Others define a person as a cancer survivor from the time of diagnosis [24,51,52], allowing individuals in phases in and out of cancer treatment to be explored together, even though the SCNs in these phases are not the same [7]. Compared to people in treatment, in those who have completed treatment, acute problems are no longer a priority, with long-term side effects and the psychosocial problems involved in the process of returning to a “normal” life taking predominance. Therefore, greater consensus on what we mean by cancer survivor would be especially useful for the advancement of research. In line with the European Organisation for Research and Treatment of Cancer [EORTC]’s [53] proposal, shared by other authors [8,13], we consider it convenient to define “cancer survivor” as a person who has completed primary cancer treatment with curative intent and is free of disease.

On the other hand, it is also worth noting the need to take into account that individuals’ cancer experiences can differ significantly, being influenced by cultural, socioeconomic, political, national, global and healthcare system factors [20,21,22,23]. It is with this last issue in mind that national-level studies are advisable [47,54].

Given the above and the lack of studies on SCNs in Spain, the present study aimed to explore the unmet SCNs of Spanish adult cancer survivors, trying to overcome some of the previous studies’ limitations. For this purpose, a multicentre approach was employed to explore the prevalence of SCNs in a large and heterogeneous sample of survivors using an assessment instrument specifically designed for this population. The prevalence of SCNs was analysed by total, domains and individually. Finally, various sociodemographic and cancer-related variables (age, gender, relationship status, employment status, primary treatments, AHT, cancer type and time since the end of primary treatment) were considered as potential moderating variables.

## 2. Materials and Methods

### 2.1. Procedure and Participants

This cross-sectional study is part of a larger project on HRQOL in adult cancer survivors and was approved by the Ethics Committees of the participating medical institutions and patient associations. A total of 4 hospitals, 2 medical centres and 7 cancer patient associations participated, all of them located in eastern and southeastern Spain, specifically in the Valencian Community and the Castilla–La Mancha regions. Eligible participants were cancer survivors who (i) were 18 years or older at the time of diagnosis, (ii) were disease-free (no evidence of disease), and (iii) had completed primary treatment with curative intent at least one month prior to recruitment, regardless of whether they were receiving adjuvant hormone therapy or the timing of its initiation or completion. Information on ongoing adjuvant hormone therapy was collected but was not used as an inclusion or exclusion criterion. In each centre, healthcare professionals invited all eligible survivors to participate, with no additional selection criteria applied. Recruitment occurred between 2017 and 2022. The questionnaire was self-administered. Participants either completed it at the centre or were provided with a prepaid envelope so that they could fill it out at home and return it by post. In the latter case, they were contacted by phone if we had not received the evaluation protocol within approximately two weeks, thereby minimising potential bias due to requiring completion at a single time point or to forgetfulness.

### 2.2. Measures

Sociodemographic and cancer-related data were collected using a set of ad hoc questions. SCNs were assessed using the CaSUN questionnaire, which evaluates cancer-related needs experienced in the previous month [55], and it has been specifically designed for cancer survivors. The instrument includes 35 items that respondents rate as follows: not needed/not applicable, met need or unmet need as weak, moderate or strong. The Spanish version of CaSUN has shown satisfactory psychometric properties [56]. It supports the use of a hierarchical model composed of a total score and scores in five domains: comprehensive care and information (9 items), physical effects (4 items), psychological effects (11 items), practical issues (6 items) and interpersonal relationships (5 items). Responses were dichotomised in terms of met needs (not need/not applicable, met need or weak unmet need) and unmet needs (moderate or strong unmet need), and the total score, the score in each domain and the specific needs were explored. To facilitate comparison, given the different lengths of the subscales, the average prevalence of needs at the total level and by domain was used. The internal consistency in the present study was satisfactory (Cronbach’s α = 0.94 for total needs, 0.88 for information, 0.70 for physical, 0.91 for psychological, 0.77 for practical and 0.81 for interpersonal domains).

### 2.3. Data Analysis

Descriptive statistics summarised sociodemographic, cancer-related and SCN data in the total sample. Several multivariate analyses of variance (MANOVAs) were executed to test differences in sociodemographic and cancer-related variables in the SCNs’ domains. Finally, frequencies and percentages were calculated to explore the prevalence of specific needs in different subgroups. Statistical significance was set at *p* ≤ 0.05, and analyses were performed using IBM SPSS Statistics, version 22.0.

## 3. Results

### 3.1. Total Sample

A total of 2271 cancer survivors were approached, with 1862 (82%) eligible and consenting to participate. The participants were aged 18 to 92 (mean age: 59.2; SD = 12.2), with a majority being over 45 (87.4%), female (58.9%), married or living with a partner (72.8%), without a university education (69.3%), and not active in the labour market (56.2%). The most common cancer diagnoses were breast (37.0%), prostate (15.9%) and colorectal (14.0%). Regarding primary treatment, 54.5% had received a primary treatment protocol that included chemotherapy. AHT was not prescribed as maintenance therapy for more than two-thirds of the patients (73.8%). Lastly, the mean time after the completion of primary treatment was 4.5 years (range: 1 month to 30 years). In total, 35% had completed it at least 5 years before the moment of assessment (long-term survivors), and 65% were short-term survivors: 23% were still in the first year after completion of primary treatment (Table 1).

The average prevalence of needs was nine (mean = 8.69; SD = 8.25), with almost half of such needs (*n* = 4) belonging to the comprehensive care and information domain. Psychological needs averaged two, practical needs one, and interpersonal and physical needs less than one (see Table 2). Half of the needs (*n* = 18) pertaining to the different domains were present in at least 20% of the participants. Among them, the need for help to manage side effects, the need for reducing stress, concern about cancer recurrence, the need for emotional support and the need for financial support and advice were present in one out of four survivors. In addition, those pertaining to the comprehensive care and information domain were present in one-third (local health service, better medical care, understandable information and complementary therapy) and even in one-half (having complaints appropriately addressed, managing health with the team and talking between doctors) of participants (see Table 3).

### 3.2. Moderators

The MANOVA results showed significant differences in SCNs as a function of all the modulating variables considered: cancer type, age, gender, relationship status, employment status, primary treatments, time since the end of primary treatment and AHT (*p* ≤ 0.001 in all cases). Differences were also found in all domains, with only two exceptions (comprehensive care and information domain as a function of hormone therapy and relationships domain as a function of relationship situation) (Table 2).

Age. Differences were found among the three age subgroups (≤45, 46–64 and ≥65 years) in all domains in that younger age was accompanied by a higher prevalence of SCNs (Table 2). In terms of specific needs (Table 3), the results showed that increasing age was accompanied by a progressive decrease in the prevalence of practically all SCNs. The vast majority of SCNs (*n* = 33) were present in at least 20% of survivors ≤45 years; they declined to slightly more than half (*n* = 23) in the 46–64 years subgroup and dropped to 9 in the ≥65 years subgroup. The most prevalent SCNs in all age subgroups were those belonging to the comprehensive care and information domain. Those linked mainly to emotional impact, as well as some linked to practical issues (e.g., financial support) and the impact on relationships (e.g., support from a partner/family and sex life), were responsible for the highest prevalence of SCNs in the younger subgroups, particularly in the youngest.

Gender. Women reported a higher prevalence of SCNs than men in all domains (Table 2). At the level of specific needs (Table 3), the higher prevalence of needs in women was particularly explicit in the physical (e.g., manage side effects and changes to quality of life), psychological (e.g., stress in my life, emotional support and talking to others) and relationship (e.g., support partner/family and impact on relationships) domains.

Relationship status. Survivors without a partner showed a higher prevalence of SCNs than those with a stable partner in all domains except for the interpersonal one (Table 2). At the level of specific needs (Table 3), those without a partner showed a higher demand for help to manage the physical effects (e.g., manage side effects and changes to quality of life) and the psychological impact (e.g., move on with my life, decisions about my life and make my life count) of cancer.

Employment status. Differences were found in all domains of SCNs according to employment status (Table 2). Post hoc comparisons showed that the sick leave and unemployed subgroups showed the highest prevalence of SCNs. At the level of specific needs (Table 3), the results showed the same pattern in terms of the number of SCNs in at least 20% of the participants and highlighted three aspects: (i) the vast majority of SCNs reached such prevalence in the sick leave (*n* = 34) and unemployed (*n* = 31) subgroups; (ii) the usually elevated range of prevalence of SCNs belonging to the domain of comprehensive care and information needs was especially high in the sick leave subgroup (61–76%); and (iii) more than half of the unemployed survivors reported employment and financial support as SCNs.

Primary treatment. Differences were found in all domains of SCNs as a function of primary treatment. The mean pattern of results indicated a higher prevalence of SCNs in the subgroups in which the primary treatment included chemotherapy (Table 2). This pattern was also repeated at the level of prevalence of specific needs (Table 3). Apart from those pertaining to the comprehensive care and information domain, only three needs (concerns about cancer coming back, financial support and ongoing case manager) reached a prevalence of 20% in the subgroup that did not receive chemotherapy. In contrast, in the subgroups that did receive it, this value was multiplied by five or six and included physical, psychological and relationship needs.

Adjuvant hormone therapy (AHT). The comprehensive care and information domain was the only one that did not show differences according to the presence or absence of AHT (Table 2). In the remaining domains, the subgroup with AHT showed a higher prevalence of SCNs. At the level of specific needs (Table 3), this subgroup also reported more need for help in the management of side effects, the psychological impact of cancer (e.g., move on with life and make life count) and its impact on relationships (e.g., support partner/family and problems with sex life).

Type of cancer. The results indicated that the haematological subgroup showed the highest prevalence of SCNs in virtually all domains. The exceptions applied only to the breast and gynaecological subgroups. The haematological subgroup only differed from the gynaecological subgroup in the comprehensive care and information and practical domains and did not differ from the breast subgroup in the psychological domain. The breast and gynaecological subgroups also showed a higher prevalence of SCNs in the different domains than most of the remaining subgroups, including the colorectal, head and neck, melanoma, and prostate subgroups. In terms of specific needs, the results also showed clear differences in their prevalence according to the diagnostic subgroups (Table 3). In the haematological subgroup, practically all of the 35 SCNs assessed reached a prevalence of 20%. Moreover, many of them were present in one-third of the participants, and the particularly high prevalence of comprehensive care and information needs (ranging from 39% to 77%) stands out. In the breast and gynaecological subgroups, the number of SCNs present in at least 20% of the participants was also particularly high (*n* = 27/28), highlighting the high physical, psychological and relationship impact of cancer compared to the other diagnostic subgroups. In the latter, SCNs with a prevalence of at least 20% were largely confined to the nine needs corresponding to the comprehensive care and information domain.

Time since the end of primary treatment. All the domains showed differences according to the time elapsed since the end of treatment (Table 2). The needs related to the comprehensive care and information domain showed differences between the subgroup in the first year after treatment ended and the other two. Physical and practical needs differed between short- and long-term survivors. Finally, psychological and interpersonal needs showed differences among the three subgroups, with a progressive decrease over time. At the level of specific needs (Table 3), it is worth noting that those present in at least 20% of the survivors experienced a notable decrease across the phases linked to the psychological and interpersonal domains. All the needs of the comprehensive care and information domain were present in at least one in five long survivors. This was also the case for those related to the need for support to manage the side effects of cancer and its treatment; the need for emotional support, in particular to manage the fear of recurrence; and also the need for support and financial advice.

## 4. Discussion

The first objective of the study addressed the prevalence of unmet SCNs among cancer survivors in Spain. The results showed a high prevalence of SCNs: approximately half of the needs assessed by the CaSUN (including needs from all domains) were present in one in five participants. The most important aspect of the guidelines for survivorship care developed by the American Cancer Society [57] and also endorsed in Spain by the Sociedad Española de Oncología Médica [58] is the recommendation for continuous assessment and management of the physical and psychosocial effects of cancer and related treatments. However, the high prevalence of unmet needs reported by the participants in our study indicates that much remains to be done.

Consistent with research identifying them as particularly prominent SCNs [8,11,14,19], the most frequently reported needs by participants were those related to comprehensive care and information. In particular, those related to the format of healthcare (local healthcare services, more active roles, attention to complaints and increased communication between healthcare professionals) were mentioned by half or more of the participants. The very high proportion of Spanish survivors who perceive gaps in this domain is a particularly relevant finding for health professionals and healthcare policymakers in our country. In this regard, cooperation between the oncologist and the primary care physician [59] should be encouraged to fill this gap. Indeed, providing informational resources to help primary care physicians support and engage with cancer survivors in their transition back to usual care is a recommendation included in the aforementioned guidelines on survivorship care [57,58]. Nevertheless, greater attention to individual SCNs, regardless of the healthcare professional providing it, could also be a relevant factor in mitigating these needs [60,61].

Highlighting both the physical and psychosocial problems faced by cancer survivors, other SCNs were also prevalent, although to a lesser extent (one in four), among study participants. In line with previous studies [8,15,16], the demand for help in managing the multiple long-term side effects of cancer treatments was one of the prevalent needs. The cancer survivors also expressed the need for help in managing the psychological impact of the disease (emotional support and stress) and most particularly the fear of cancer recurrence. Regarding the psychological impact of cancer experience, fear of cancer recurrence is considered the most common SCN among cancer survivors [9,10]. Finally, among psychological needs, the need to talk to other survivors—reported by one in five participants—is also noteworthy. Away from the focus on the medical treatment itself, the survivorship phase often involves processing the cancer experience [62]. Talking to others in the same circumstances could be explained by the attempt to assimilate and normalise one’s past and present situation. Indeed, the need for opportunities to foster empathy and share experiences is one of the most prevalent SCNs among cancer survivors, according to a review by Tabari-Khomeiran et al. [16].

Consistent with the research results [25,26], the need for financial support and advice also emerged as a prevalent need reported by one out of four participants. It is remarkable that in a country like Spain, with a national public healthcare system, economic problems continue to be so relevant. It could be argued in this regard that, although healthcare costs are covered, the survivor has to face other economic issues that may arise from the impact of cancer in the work and legal spheres. Previous studies in our country point out that 45% of working-age survivors do not return to work [63]. This aligns with the recommendations of international organisations regarding the inclusion of such issues in comprehensive cancer survivorship care [1,2]. The social sector should play a complementary role to that of the health sector to improve the reintegration of cancer survivors into normal social functions and activities without discrimination [64]. In this context, laws such as the Right to Be Forgotten [65] are particularly relevant in improving the current global situation faced by cancer survivors.

A second objective focused on different sociodemographic and disease-related variables as possible relevant determinants of the SCNs among cancer survivors. With only two exceptions (the comprehensive care and information domain as a function of hormone therapy and the relationships domain as a function of relationship status), all the variables explored were shown to be modulators of the presence of SCNs in the different domains explored. The same pattern was replicated on average when the specific prevalence of each need was considered. The risk profile regarding the presence of SCNs pointed to younger age; being female; not having a partner; being on medical leave and/or unemployed; having a diagnosis of haematological, breast or gynaecological cancer; having received systemic treatment (chemotherapy and/or hormone therapy); and being at an earlier stage of survival.

The prevalence of unmet needs decreased progressively across the age subgroups. In fact, among participants aged 65 years or older, the needs present in at least one in five participants were exclusively those belonging to the comprehensive care and information domain. Our results thus support those obtained in the literature regarding the higher prevalence of SCNs in younger adult cancer survivors [7,27,30,31,32]. When the cancer experience occurs earlier in adulthood, the lesser life experience/trajectory of the individual is accompanied by the greater disruption that the disease causes to personal and professional development [27]. Consequently, a greater need for help in managing the disease’s broad impact on multiple aspects of life is to be expected.

Consistent with the literature [7,27,31,36], women exhibited a higher prevalence of unmet care needs compared to men. It seems appropriate to appeal to the different biological and psychosocial profiles that characterise women and men [66,67] rather than merely discount a genuinely worse female oncologic experience [68].

Studies have traditionally highlighted the important role of the partner as a source of social support in the cancer experience and thus associated it with better adjustment [69,70] and a lower presence of SCNs [27,37,38,71]. In line with these data, our results showed the lowest prevalence of SCNs among those survivors who had a stable partner.

Several studies highlight the importance of being laborally active after the cancer experience as a way of returning to everyday life to the extent that this allows professional and personal development, which in turn may promote greater social support, contributing to the improvement of the survivor’s quality of life and emotional adjustment [64,72,73]. Supporting this idea and consistent with research results [39,40], among working-age participants, the active subgroup reported the lowest prevalence of SCNs. Only the subgroup on sick leave reported more than the unemployed subgroup, which is understandable when considering that, in addition to their unemployment situation, they may have medical conditions that account for their sick leave.

Compared to other local treatments, chemotherapy treatment causes greater side effects [74]. On the other hand, the side effects of AHT, although less severe, remain longer since the treatment period with AHT is considerably longer [75]. Consequently, both treatments can severely affect the quality of life (QoL) and wellbeing of cancer survivors [27,71,76,77,78,79]. In this regard, and consistent with existing results in the literature [11,40,42,43], data from this study support a higher prevalence of SCNs among survivors who had been prescribed chemotherapy or AHT.

The type of cancer-specific analysis also provided nuanced insights. Among the different types of cancer that, according to the literature, represent a higher risk of SCNs, our results highlighted haematological, gynaecological and breast cancer. Research findings support the association between the latter two types of cancer and increased prevalence of SCNs [40,41]. The haematological type has been scarcely studied in the past due to its poor prognosis. However, survival rates and quality of life have improved significantly over the last few decades [80], and this subgroup requires specific attention. Several aspects related to the treatment and prevalence profile of these cancers may account for the results obtained. Standard treatment options in haematological cancer (bone marrow transplantation, peripheral blood cell transplantation and high-dose chemotherapy) are lengthy, invasive and often lead to debilitating side-effects, including extensive comorbidities and severe adverse drug reactions [81,82,83,84]. Age may be another explanation of the results. Two facts stand out in this regard: (i) younger age is associated with prevalence of haematological and gynaecological diagnoses [85,86] and (ii) a higher percentage of new diagnoses in people aged 65 years or older occurred in men compared to women [2]. The higher prevalence of SCNs in the breast and gynaecological subgroups could also be because these two types of cancer are specific to the female sex. Thus, both psychosocial and biological issues previously discussed could be behind the results obtained. One last factor to consider, given the high percentage of hormone receptor-positive breast cancer patients [87], is the widespread prescription of AHT in the breast subgroup, which may influence the higher prevalence of SCNs in this subgroup.

Consideration of the time elapsed since the end of the primary treatment revealed significant and distinct evolutions depending on the SCN domain considered. Exceeding the first-year post-treatment period led to a decrease in the comprehensive care and information domain-related needs. Psychological and interpersonal needs progressively decreased through the different phases, and reaching long-term survival marked a decrease in physical and practical needs. Notwithstanding these significant changes, the prevalence of SCNs remained remarkable even several years after the completion of primary treatment. In particular, the needs previously highlighted as most prevalent (medical information and care, side effects, psychological impact, fear of recurrence, and financial support and advice) were still present in at least 20% of long survivors.

In summary, our study emphasises the importance of enhancing health service delivery to meet the diverse care needs of cancer survivors. Enhancing communication among healthcare providers, optimising (and making more visible) care coordination and integrating survivorship care plans into routine practice are essential steps towards improving survivorship outcomes. Addressing these gaps, where assigning a role to the primary care physician can be key, could both improve the efficiency of healthcare and contribute to the overall wellbeing of cancer survivors. Other highly prevalent needs should also be addressed, and the sociodemographic profile should be taken into account, as well as different variables related to the disease, in order to establish risk subgroups in which to prioritise care. Despite the valuable insights gained—and considering that this is the first study conducted in our country on this topic—several limitations should be taken into account when interpreting the results. Firstly, despite the large size of the sample studied, the size of some of the subgroups established according to the type of cancer was limited and some diagnoses were left out of focus, such as lung cancer, which in recent years has experienced a very significant increase according to survival figures [1,2]. It would be advisable for future studies to contemplate larger samples of some types of cancer and also include others that have been less contemplated up to now. Secondly, we did not explore possible interactions between the different risk factors, which would be advisable in future studies. It is possible, for example, that the role of the partner as a source of social support may vary according to gender. Thirdly, the advisable circumscription of the study at the national level, given the differences in health systems across countries, impacts the generalizability of our findings beyond the specific context of Spanish adult cancer survivors. Future research should aim to replicate these findings in other cultural and healthcare settings to validate their applicability and robustness. Comparative studies across different countries or healthcare systems would also provide valuable insights into the factors influencing variations in SCNs and outcomes. By examining how cultural, socioeconomic and healthcare system factors interact with different sociodemographic and disease-related variables to shape survivorship experiences, researchers can identify best practices and policy recommendations for improving survivorship care globally. Another limitation of our study, particularly regarding the temporal evolution of care needs, is its cross-sectional design, which does not allow for the examination of cause–effect relationships or changes over time. In this sense, prospective and longitudinal studies would be of great value.

Notwithstanding the limitations of the study, its strengths include (i) its multicentre nature, which incorporates a large and heterogeneous sample of survivors; (ii) the use of a standardised instrument such as the CaSUN questionnaire, specifically created for the assessment of the various supportive care needs of cancer survivors and with satisfactory psychometric properties; and (iii) the exploration of unmet care needs at both the domain and specific levels. To minimise selection and information bias, several measures were implemented. The study was conducted in a multicentre setting, involving both hospitals and patient associations, to ensure a heterogeneous and diverse sample. All eligible survivors were invited to participate, regardless of cancer type. Telephone follow-ups were carried out to remind participants to return the completed assessment protocol, thereby reducing potential bias related to timing or forgetfulness. Finally, a large sample size was obtained to strengthen data validity and further minimise potential bias. Consequently, the findings contribute valuable insights into the diverse SCNs of cancer survivors and underscore the importance of personalised, holistic approaches to care. By addressing these implications and pursuing future research directions, healthcare providers and policymakers can work towards improving the quality of life and wellbeing of cancer survivors across the continuum of survivorship.

## 5. Conclusions

The study highlights significant SCNs among cancer survivors in Spain, with considerable variation across different sociodemographic (age, gender, partner and employment status) as well as cancer-related variables (type of cancer, treatment and stage of survival). Transversal to the different subgroups emerged comprehensive care and information (the most prevalent domain of unmet needs), followed by specific needs such as managing side effects, psychological impact, fear of recurrence, and financial support and advice. The highest burdens of unmet needs are found in those of younger age; females; those who do not have a partner; those who are on medical leave and/or unemployed; those with a diagnosis of haematological, breast or gynaecological cancer; those who have received systemic treatment (chemotherapy and/or AHT); and those at an earlier stage of survival as special risk groups. Future interventions should focus on enhancing communication, coordination and the overall quality of care to improve the wellbeing and quality of life of cancer survivors.

## Figures and Tables

**Table 1:** Characteristics of the participants (*N* = 1864).

Variable		*n*	%
**Age**			
(*N* = 1864; mean: 59.16	18–45 years	235	12.6
*SD* = 12.18; range = 18–92)	46–64 years	940	54.4
	≥65 years	690	37.0
			
Gender	Female	1090	58.9
(*n* = 1851)	Male	761	41.1
			
Relationship status (*n* = 1829)	With partner	1332	72.8
	Single	497	27.2
			
Level of studies (*n* = 1773)	No studies	130	7.3
	Primary education	721	40.7
	Secondary education	377	21.3
	Higher education	545	30.7
			
Employment status (*n* = 1798)	Employed	503	28.0
	Unemployed	164	9.1
	On sick leave	106	5.9
	Pre-retired/retired	741	41.2
	Housework	167	9.3
	Other	117	6.5
			
Cancer site (*n* = 1829)	Breast	677	37.0
	Prostate	291	15.9
	Colorectal	256	14.0
	Haematological	103	5.6
	Head and neck	104	5.7
	Gynaecological	97	5.3
	Melanoma	79	4.3
	Multiple	86	4.7
	Others	136	7.4
		
Primary treatments (*n* = 1.865)	S, RT or S + RT	721	45.6
	CT or CT + S	270	17.1
	CT + RT or CT + RT + S	591	37.4
			
Time since the end of primary treatment (*n* = 1829; mean = 53.53; *SD* = 59.95; range: 1–360 months)	≤12 months	413	22.6
	>12 months–<5 years	774	42.3
	≥5 years	642	35.1
			
AHT	YES	480	26.2
	NO	1349	73.8

*Note:* S = Surgery, RT = Radiotherapy, CT = Chemotherapy.

**Table 2:** MANOVA results showing mean CaSUN domain scores for the total sample and across sociodemographic and cancer-related subgroups.

	QLACS Domains				
	Comprehensive Care and Information	Physical Effects	Psychological Effects	Practical Issues	Relationships
	Mean (SD)	Mean (SD)	Mean (SD)	Mean (SD)	Mean (SD)
***Total sample***	4.02 (3.14)	0.68 (1.07)	2.06 (3.10)	1.11 (1.56)	0.83 (1.39)
(mean= 8.69; SD = 8.25)	F, mean (SD)	F, mean (SD)	F, mean (SD)	F, mean (SD)	F, mean (SD)
***Age*** (Pillai’s Trace = *0.111*; *F* = *21.81, df between* = *10*; *df error* = *3718*; *p* ≤ *0.001*)	F = 34.03 ***	F = 90.31 ***	F = 74.63 ***	F = 72.43 ***	F = 58.84 ***
≤*45 years*	5.11 (2.96) ^b,c^	1.28 (1.35) ^b,c^	3.57 (3.78) ^b,c^	1.71 (1.88 ^b,c^)	1.46 (1.70) ^b,c^
*46*–*64 years*	4.25 (3.16) ^a,c^	0.80 (1.10) ^a,c^	2.40 (3.25) ^a,c^	1.34 (1.65) ^a,c^	0.95 (1.47) ^a,c^
≥*65 years*	3.34 (3.03) ^a,b^	0.32 (0.74) ^a,b^	1.07 (2.19) ^a,b^	0.58 (1.01) ^a,b^	0.44 (1.00) ^a,b^
					
***Gender*** (Pillai’s Trace = *0.068*; *F* = *586.22, df between* = *5.0*; *df error* = *1823*; *p* ≤ *0.001*)	F = 10.40 ***	F = 69.90 ***	92.00 ***	37.80 ***	18.51 ***
*Female*	4.22 (3.17)	0.85 (1.14)	2.62 (3.34)	1.29 (1.64)	0.94 (1.47)
*Male*	3.74 (3.08)	0.44 (0.89)	1.25 (2.51)	0.84 (1.39)	0.66 (1.26)
					
***Relationship status*** (Pillai’s Trace = *0.028*; *F* = *10.50*, *df between* = *5.0*; *df error* = *1844*; *p* ≤ *0.001*)	F = 15.35 ***	F = 25.08 ***	F= 22.39 ***	F = 18.39 ***	F = 2.13
*Single/divorced/widowed*	4.46 (3.12	0.87 (1.19)	2.58 (3.38)	1.34 (1.70)	0.88 (1.38)
*Married/with partner*	3.82 (3.12)	0.59 (0.99)	1.82 (2.91)	0.99 (1.45)	0.78 (1.36)
					
***Employment status*** (Pillai’s Trace = *0.219*; *F* = *23.70*, *df between* = *15.0*; *df error* = *4524*; *p* ≤ *0.001*)	F = 20.62 ***	F = 47.09 ***	F = 69.54 ***	F = 92.40 ***	F = 41.59 ***
*Active*	4.21 (3.09) ^f,g^	0.69 (1.08) ^e,f,g^	1.90 (2.87) ^e,f,g^	0.97 (1.45) ^e,f,g^	0.70 (1.34) ^e,f,g^
*Unemployed*	4.23 (3.12) ^f^	1.05 (1.25) ^d,f,g^	3.05 (3.44) ^d,f,g^	2.30 (1.78) ^d,f,g^	1.15 (1.55) ^d,f,g^
*On sick leave*	5.93 (2.91) ^d,e,g^	1.46 (1.24) ^d,e,g^	5.09 (3.91) ^d,e,g^	2.33 (2.00) ^d,e,g^	1.96 (1.77) ^d,e,g^
*Retired/pre-retired*	3.55 (3.04)	0.41 (0.82) ^d,e,f^	1.24 (2.36) ^d,e,f^	0.66 (1.15) ^d,e,f^	0.54 (1.14) ^d,e,f^
					
***Primary treatments*** (Pillai’s Trace = *0.074*; *F* = *13.94*, *df between* = *10.0*; *df error* = *3620*; *p* ≤ *0.001*)	F = 18.35 ***	F = 51.61 ***	F = 26.54 ***	F = 40.40 ***	F = 17.11 ***
S, RT or S + RT	3.61 (3.09) ^j,i^	0.41 (0.83) ^j.i^	1.48 (2.65) ^j,i^	0.74 (1.28) ^j,i^	0.62 (1.18) ^j,i^
CT or CT + S	4.84 (3.06) ^h.j^	0.92 (1.22) ^h^	2.72 (3.48) ^h^	1.43 (1.76) ^h^	1.09 (1.56) ^h^
CT + RT or CT + RT + S	4.11 (3.14) ^h,i^	0.68 (1.06) ^h^	2.40 (3.24) ^h^	1.36 (1.65) ^h^	0.93 (1.47) ^h^
					
***Hormonotherapy*** (Pillai’s Trace = *0.014*; *F* = *5.19*, *df between* = *5.0*; *df error* = *1823*; *p* ≤ *0.001*)	F = 0.000	F = 14.57 ***	F = 16.87 ***	F = 8.80 **	F = 8.52 **
*Yes*	4.01 (3.22)	0.84 (1.16)	2.54 (3.30)	1.27 (1.66)	0.98 (1.50)
*No*	4.01 (3.11)	0.62 (1.02)	1.87 (2.98)	1.03 (1.50)	0.76 (1.33)
					
***Type of cancer*** (Pillai’s Trace = *0.153*; *F* = *8.42*, *df between* = *30*; *df error* = *8000*; *p* ≤ *0.001*)	F = 6.60 ***	F = 23.65 ***	F = 23.11 ***	F = 23.64 ***	F = 13.99 ***
*Breast*	4.26 ^o,q^(3.18)	0.90 ^o,p,q,r,t^(1.16)	2.71 ^o,p,r,t^(3.37)	1.37 ^o,p,q,r,t^(1.66)	0.98 ^o,p,q,t^(1.49)
*Prostate*	3.32 ^n,q^ (3.11)	0.31 ^n,q,s^ (0.71)	0.87 ^n,q,s^ (2.00)	0.50 ^n,q,s^ (0.95)	0.63 ^n,q,s^ (1.18)
*Colorectal*	4.01 ^q^ (2.94)	0.46 ^n,q,s^ (0.96)	1.40 ^n,q,s^ (2.67)	0.86 ^n,q^ (2.05)	0.57 ^n,q,s^ (1.22)
Haematological	5.37 ^n,o,p,r,t^ (2.88)	1.29 ^n,o,p,r,t^ (1.30)	3.64 ^o,p,r,t^ (3.81)	2.16 ^n,o,p,r,s,t^ (2.05)	1.67 ^n,o,p,r,t^ (1.77)
Head and neck	3.70 ^q^ (3.15)	0.53 ^n,q^ (0.90)	1.35 ^n,q,s^ (2.41)	0.81 ^n,q^ (1.18)	0.54 ^q,s^ (1.10)
Gynaecological	4.04 ^q^ (3.36)	0.96 ^o,p,r,t^ (1.19)	2.78 ^o,p,r,t^ (3.49)	1.09 ^o,q^ (1.46)	1.19 ^o,p,r,t^ (1.64)
Melanoma	3.75 (3.06) ^q^	0.20 ^n,q,s^ (0.63)	1.14 ^n,q,s^ (2.33)	0.58 ^n,q^ (1.27)	0.30 ^n,p,q,s^(0.93)
					
***Time since the end of primary treatment treatments*** (Pillai’s Trace = *0.028*; *F* = *5.20*, *df between* = *10.0*; *df error* = *3646*; *p* ≤ *0.001*)	F = 8.66 ***	F = 11.95 ***	F = 14.73 ***	F = 4.95 ***	F = 18.08 ***
≤12 months	4.59 (3.25) ^l,m^	0.84 (1.13) ^m^	2.71 (3.51) ^l,m^	1.23 (1.56) ^m^	1.15 (1.59) ^l,m^
>12 months–<5 years	3.94 (3.12) ^k^	0.73 (1.13) ^m^	2.07 (3.08) ^k,m^	1.17 (1.59) ^m^	0.83 (1.39) ^k,m^
≥5 years	3.80 (3.07) ^k^	0.53 (0.94) ^k,l^	1.66 (2.78) ^k,l^	0.96 (1.51) ^k,l^	0.63 (1.24) ^k,l^

Note: *** *p* ≤ 0.001. S = Surgery, RT = Radiotherapy, CT = Chemotherapy. a = ≤45 years; b = 46–64 years; c = ≥65 years; d = active; e = unemployed; f = sick leave; g = retired/pre-retired; h = S, RT or S + RT; i = CT or CT + S; j = CT + RT or CT + RT + S; k = ≤ 12 months; l = >12 months–<5 years; m = ≥5 years; n = breast; o = prostate; p = colorectal; q = haematological; r = head and neck; s = gynaecological; t = melanoma.

**Table 3:** Frequencies and percentages of specific needs (CaSUN) for total sample and by sociodemographic and cancer-related variables.

	**Total Sample**	**Age**							**Gender**					**Relationship Status**			
	***n* (%)**	**≤45 Years** ***n* (%)**			**46–64 Years** ***n* (%)**		**≥65 Years** ***n* (%)**		**Women** ***n* (%)**		**Men** ***n* (%)**			**Single** ***n* (%)**			**With Partner** ***n* (%)**
**Comprehensive Care and Information**																	
1. Up-to-date information	568 (30.5)	110 (46.8)			306 (32.6)		152 (22.0)		365 (33.5)		198 (26.0)			176 (35.4)			375 (28.2)
2. Information for others	460 (24.7)	89 (37.9)			234 (24.9)		137 (19.9)		274 (25.1)		183 (24.0)			121 (24.3)			323 (24.2)
3. Understandable information	786 (42.1)	127 (54.0)			422 (44.9))		237 (34.3)		481 (44.1)		299 (39.3)			232 (46.7)			532 (39.9)
4. Best medical care	835 (44.8)	129 (54.9)			457 (48.6)		249 (36.1)		518 (47.5)		313 (41.1)			256 (51.5)			559 (42.0)
5. Local healthcare service	900 (48.3)	138 (58.7)			467 (49.7)		295 (42.8)		548 (50.3)		345 (45.3)			267 (53.7)			610 (45.8)
6. Manage health with team	1065 (57.1)	163 (69.4)			547 (58.2)		355 (51.4)		633 (58.1)		426 (56.0)			319 (64.2)			722 (54.2)
7. Doctors talk to each other	1017 (54.5)	150 (63.8)			542 (57.7)		325 (47.1)		610 (58.0)		401 (52.7)			294 (59.2)			701 (52.6)
8. Complaints addressed	1079 (57.9)	160 (68.1)			567 (60.3)		352 (51.0)		634 (58.2)		438 (57.6)			313 (63.0)			741 (55.6)
9. Complimentary therapy	787 (42.2)	135 (57.4)			450 (47.9)		202 (29.3)		536 (49.2)		245 (32.2)			239 (48.1)			524 (39.3)
**Physical effects**																	
11. Manage side effects	460 (24.7)	88 (37.4)			281 (29.9)		91 (13.2)		342 (31.4)		115 (15.1)			155 (31.2)			291 (21.8)
12. Changes to quality of life	419 (22.5)	92 (39.1)			255 (27.1)		72 (10.4)		318 (29.2)		98 (12.9)			140 (28.2)			266 (20.0)
13. Fertility	118 (6.3)	51 (21.7)			49 (5.2)		18 (2.6)		57 (5.2)		59 (7.8)			40 (8.0)			70 (5.3)
26. Changes to my body	271 (14.5)	69 (29.4)			165 (17.6)		37 (5.4)		209 (19.2)		60 (7.9)			97 (19.5)			164 (12.3)
**Psychological effects**																	
10. Reduce stress in my life	486 (24.7)	107 (45.5)			296 (31.5)		83 (12.0)		374 (34.3)		109 (14.3)			157 (31.6)			313 (23.5)
19. Concerns about cancer coming back	471 (25.3)	87 (37.0)			269 (28.6)		115 (16.7)		331 (30.4)		138 (18.1)			140 (28.2)			316 (23.7)
20. Emotional support for me	465 (24.9)	104 (44.3)			262 (27.8)		99 (14.3)		359 (32.9)		104 (13.7)			164 (33.0)			289 (21.7)
24. Talking to others	416 (22.3)	82 (34.9)			239 (25.4)		95 (13.8)		294 (27.0)		121 (15.9)			134 (27.0)			269 (20.2)
29. Move on with my life	291 (15.6)	61 (26.0)			186 (19.8)		44 (6.4)		222 (20.4)		68 (8.9)			99 (19.9)			182 (13.7)
30. Changes to beliefs	361 (19.4)	81 (34.5)			213 (22.7)		67 (9.7)		273 (25.0)		86 (11.3)			115 (23.1)			232 (17.4)
31. Acknowledging the impact	305 (16.4)	77 (32.8)			175 (18.6)		53 (7.7)		226 (20.7)		78 (10.2)			106 (21.3)			191 (14.3)
32. Survivor expectations	308 (16.5)	76 (32.3)			182 (19.4)		50 (7.2)		232 (21.3)		74 (9.7)			100 (20.1)			197 (14.8)
33. Decisions about my life	325 (17.4)	76 (32.3)			194 (20.6)		55 (8.0)		249 (22.8)		27 (26.2)			114 (22.9)			200 (15.0)
34. Spiritual beliefs	138 (7.4)	34 (14.5)			82 (8.7)		22 (3.2)		103 (9.4)		34 (4.5)			54 (10.9)			76 (5.7)
35. Make my life count	267 (14.3)	53 (22.6)			182 (17.2)		52 (7.5)		197 (18.1)		68 (8.9)			98 (19.7)			159 (11.9)
**Practical issues**																	
14. Employment	224 (12.0)	61 (26.0)			148 (15.7)		15 (2.2)		169 (15.5)		52 (6.6)			77 (15.5)			134 (10.1)
15. Financial support	538 (28.8)	94 (40.0)			334 (35.5)		110 (15.9)		373 (34.2)		160 (21.0)			168 (33.8)			354 (26.6)
16. Life/travel insurance	258 (13.8)	51 (21.7)			169 (17.9)		39 (5.7)		175 (16.1)		79 (10.4)			88 (17.7)			157 (11.8)
17. Legal services	279 (15.0)	63 (26.8)			177 (18.8)		39 (5.7)		191 (17.5)		84 (11.0)			101 (20.3)			165 (12.4)
18. Accessible hospital parking	260 (13.9)	45 (19.1)			133 (14.1)		82 (11.9)		151 (13.9)		105 (13.8)			76 (15.3)			174 (13.1)
28. Ongoing case manager	511 (27.4)	88 (37.4)			304 (32.3)		119 (17.2)		348 (31.9)		159 (20.9)			156 (31.4)			340 (25.5)
**Relationships**																	
21. Support partner/family	355 (19.0)	80 (34.0)			202 (21.5)		73 (10.6)		241 (22.1)		113 (14.8)			102 (20.5)			241 (18.1)
22. Impact on my relationship	322 (17.3)	74 (31.5)			185 (19.7)		63 (9.1)		220 (20.2)		101 (13.3)			68 (13.7)			240 (18.0)
23. New relationships	218 (11.7)	51 (21.7)			132 (14.0)		35 (5.1)		154 (14.1)		63 (8.3)			82 (16.5)			125 (9.4)
25. Handle social/work situations	271 (14.5)	63 (26.8)			164 (17.4)		44 (6.4)		186 (17.1)		84 (11.0)			98 (19.7)			161 (12.1)
27. Problems with sex life	374 (20.1)	75 (31.9)			213 (22.7)		86 (12.5)		228 (20.9)		143 (18.8)			89 (17.9)			270 (20.3)
		**Employment Status**							**Primary Treatments**								
		**Active** ***n* (%)**		**Unemployed** ***n* (%)**			**On Sick Leave** ***n* (%)**		**Retired** ***n* (%)**		**S, RT or S + RT** ***n* (%)**			**CT or CT + S** ***n* (%)**			**CT + RT or CT + RT + S** ***n* (%)**
**Comprehensive care and information**																	
1. Up-to-date information		168 (33.4)		50 (30.5)			64 (60.4)		180 (24.3)		213 (26.0)			119 (37.4)			218 (32.1)
2. Information for others		126 (25.0)		35 (21.3)			52 (49.1)		156 (21.1)		185 (22.6)			94 (29.6)			166 (24.4)
3. Understandable information		218 (43.3)		68 (41.5)			74 (69.8)		273 (36.8)		300 (36.6)			160 (50.3)			300 (44.2)
4. Best medical care		243 (48.3)		80 (48.8)			65 (61.3)		285 (38.5)		324 (39.6)			170 (53.5)			318 (46.5)
5. Local healthcare service		243 (48.3)		84 (51.2)			68 (64.2)		332 (44.8)		352 (43.0)			189 (59.4)			335 (49.3)
6. Manage health with team		296 (58.8)		96 (58.5)			78 (73.6)		398 (53.7)		439 (53.6)			218 (68.6)			381 (56.1)
7. Doctors talk to each other		285 (56.7)		88 (53.7)			81 (76.4)		373 (50.3)		421 (51.4)			200 (62.9)			372 (54.8)
8. Complaints addressed		1079 (57.9)		103 (62.8)			77 (72.6)		399 (53.8)		459 (56.0)			222 (69.8)			371 (54.6)
9. Complimentary therapy		232 (46.1)		89 (54.3)			70 (66.0)		233 (31.4)		264 (32.2)			166 (52.2)			331 (48.7)
**Physical effects**																	
11. Manage side effects		124 (24.7)		61 (37.2)			51 (48.1)		123 (16.6)		123 (15.0)			95 (29.9)			232 (34.2)
12. Changes to quality of life		102 (20.3)		63 (38.4)			58 (54.7)		96 (13.0)		108 (13.2)			96 (30.2)			204 (30.0)
13. Fertility		46 (9.1)		15 (9.1)			8 (7.5)		25 (3.4)		44 (5.4)			28 (8.8)			42 (6.2)
26. Changes to my body		75 (14.9)		34 (20.7)			38 (35.8)		59 (8.0)		60 (7.3)			73 (23.0)			132 (19.4)
**Psychological effects**																	
10. Reduce stress in my life		147 (29.2)		61 (37.2)			55 (51.9)		104 (14.0)		154 (18.8)			115 (36.2)			201 (29.6)
19. Concerns about cancer coming back		128 (25.4)		45 (27.4)			58 (54.7)		133 (17.9)		166 (20.3)			100 (31.4)			193 (28.4)
20. Emotional support for me		124 (24.7)		51 (31.1)			60 (56.6)		118 (15.9)		157 (19.2)			103 (32.4)			193 (28.4)
24. Talking to others		105 (20.9)		53 (32.3)			48 (45.3)		42 (18.4)		138 (16.8)			91 (28.6)			177 (26.1)
29. Move on with my life		59 (11.7)		48 (28.0)			45 (42.5)		62 (8.4)		87 (10.6)			64 (20.1)			129 (19.0)
30. Changes to beliefs		90 (17.9)		49 (29.9)			51 (48.1)		79 (10.7)		113 (13.8)			79 (24.8)			157 (23.1)
31. Acknowledging the impact		78 (15.5)		42 (25.6)			49 (46.2)		71 (9.6)		96 (11.7)			73 (23.0)			125 (18.4)
32. Survivor expectations		77 (15.3)		43 (26.2)			50 (47.2)		63 (8.5)		88 (10.7)			75 (23.6)			134 (19.7)
33. Decisions about my life		73 (14.5)		55 (33.5)			57 (53.8)		67 (9.0)		94 (11.5)			73 (23.0)			144 (21.2)
34. Spiritual beliefs		24 (4.8)		14 (8.5)			26 (24.5)		30 (4.0)		36 (4.4)			33 (10.4)			64 (9.4)
35. Make my life count		51 (10.1)		41 (25.0)			41 (38.7)		67 (9.0)		86 (10.5)			60 (18.9)			111 (16.3)
**Practical issues**																	
14. Employment		42 (8.3)		86 (52.4)			32 (30.2)		15 (2.0)		48 (5.9)			50 (15.7)			117 (17.2)
15. Financial support		136 (27.0)		104 (63.4)			58 (54.7)		123 (16.6)		167 (20.4)			109 (34.3)			242 (35.6)
16. Life/travel insurance		80 (15.9)		38 (23.2)			28 (26.4)		58 (7.8)		74 (9.0)			59 (18.6)			114 (16.8)
17. Legal services		66 (13.1)		50 (30.5)			44 (41.5)		52 (7.0)		73 (8.9)			70 (22.0)			121 (17.8)
18. Accessible hospital parking		42 (8.3)		24 (14.6)			31 (29.2)		101 (13.6)		84 (10.3)			57 (17.9)			109 (16.1)
28. Ongoing case manager		120 (23.9)		77 (47.0)			54 (50.9)		141 (19.0)		163 (19.9)			109 (34.3)			221 (32.5)
**Relationships**																	
21. Support partner/family		89 (17.7)		36 (22.0)			49 (46.2)		92 (12.4)		116 (14.2)			86 (27.0)			142 (20.9)
22. Impact on my relationship		85 (16.9)		39 (23.8)			44 (41.5)		82 (11.1)		110 (13.4)			72 (22.6)			131 (19.3)
23. New relationships		51 (10.1)		31 (18.9)			35 (33.0)		54 (7.3)		59 (7.2)			49 (15.4)			100 (14.7)
25. Handle social/work situations		69 (13.7)		41 (25.0)			44 (41.5)		57 (7.7)		84 (10.3)			68 (21.4)			109 (16.1)
27. Problems with sex life		99 (19.7)		42 (25.6)			36 (34.0)		116 (15.7)		140 (17.1)			71 (22.3)			152 (22.4)
		**Time Since the End of Primary Treatment**										**AHT**					
		**≤12 Months** ***n* (%)**				**>12 Months–< 5 Years** ***n* (%)**			**≥5 Years** ***n* (%)**			**NO** ***n* (%)**			**YES** ***n* (%)**		
**Comprehensive care and information**																	
1. Up-to-date information		172 (41.6)				234 (30.2)			154 (24.0)			392 (29.1)			163 (34.0)		
2. Information for others		150 (36.3)				173 (22.4)			132 (20.6)			325 (24.1)			123 (25.6)		
3. Understandable information		206 (49.9)				316 (40.8)			252 (39.3)			556 (41.2)			212 (44.2)		
4. Best medical care		190 (48.0)				349 (45.1)			286 (44.5)			606 (44.9)			211 (44.0)		
5. Local healthcare service		213 (51.6)				366 (47.3)			309 (48.1)			664 (49.2)			216 (45.0)		
6. Manage health with team		259 (62.7)				436 (56.3)			354 (55.1)			791 (58.6)			254 (52.9)		
7. Doctors talk to each other		253 (61.3)				412 (53.2)			334 (52.0)			744 (55.2)			255 (53.1)		
8. Complaints addressed		250 (60.5)				433 (57.2)			364 (56.7)			806 (59.7)			253 (52.7)		
9. Complimentary therapy		202 (48.9)				320 (41.3)			253 (39.4)			528 (39.1)			240 (50.0)		
**Physical effects**																	
11. Manage side effects		131 (31.7)				195 (25.2)			131 (20.4)			294 (21.8)			158 (32.9)		
12. Changes to quality of life		109 (26.4)				188 (24.3)			117 (18.2)			270 (20.0)			140 (29.2)		
13. Fertility		35 (8.5)				57 (7.4)			25 (3.9)			95 (7.0)			19 (4.0)		
26. Changes to my body		73 (17.7)				127 (16.4)			68 (10.6)			181 (13.4)			85 (17.7)		
**Psychological effects**																	
10. Reduce stress in my life		119 (28.8)				209 (27.0)			153 (23.8)			316 (23.4)			157 (32.7)		
19. Concerns about cancer coming back		149 (36.1)				180 (23.3)			138 (21.5)			319 (23.6)			142 (29.6)		
20. Emotional support for me		130 (31.5)				204 (26.4)			128 (19.9)			301 (22.3)			155 (32.3)		
24. Talking to others		128 (31.0)				165 (21.3)			118 (18.4)			279 (20.7)			129 (26.9)		
29. Move on with my life		89 (21.5)				114 (14.7)			82 (12.8)			188 (13.9)			94 (19.6)		
30. Changes to beliefs		104 (25.2)				149 (19.3)			101 (15.7)			238 (17.6)			114 (23.8)		
31. Acknowledging the impact		99 (24.0)				136 (17.6)			68 (10.6)			206 (15.3)			91 (19.0)		
32. Survivor expectations		102 (24.7)				129 (16.7)			74 (11.5)			200 (14.8)			100 (20.8)		
33. Decisions about my life		94 (22.8)				145 (18.7)			82 (12.8)			204 (15.1)			111 (23.1)		
34. Spiritual beliefs		37 (9.0)				53 (6.8)			46 (7.2)			91 (6.7)			43 (9.0)		
35. Make my life count		70 (16.9)				116 (15.0)			75 (11.7)			178 (13.2)			82 (17.1)		
**Practical issues**																	
14. Employment		58 (13.6)				97 (12.5)			66 (10.3)			138 (10.2)			78 (16.3)		
15. Financial support		125 (30.3)				240 (31.0)			164 (25.5)			360 (26.7)			160 (33.3)		
16. Life/travel insurance		55 (13.3)				112 (14.5)			86 (13.4)			173 (12.8)			77 (16.0)		
17. Legal services		78 (18.9)				123 (15.9)			74 (11.5)			188 (13.9)			80 (16.7)		
18. Accessible hospital parking		58 (14.0)				116 (15.5)			80 (12.5)			178 (13.2)			74 (15.4)		
28. Ongoing case manager		140 (33.9)				217 (28.0)			144 (22.4)			354 (26.2)			144 (30.0)		
**Relationships**																	
21. Support partner/family		118 (28.6)				138 (17.8)			96 (15.0)			242 (17.9)			105 (21.9)		
22. Impact on my relationship		106 (25.7)				142 (18.3)			73 (11.4)			216 (16.0)			98 (20.4)		
23. New relationships		68 (16.5)				88 (11.4)			59 (9.2)			136 (10.1)			75 (15.6)		
25. Handle social/work situations		79 (19.1)				113 (14.6)			74 (11.5)			187 (13.9)			76 (15.8)		
27. Problems with sex life		106 (25.7)				162 (20.9)			102 (15.9)			249 (18.5)			115 (24.0)		
		**Type of Cancer**															
		**Breast** ***n* (%)**	**Prostate** ***n* (%)**			**Colorectal** ***n* (%)**		**Haematological** ***n* (%)**		**Head and Neck** ***n* (%)**			**Gynaecological** ***n* (%)**			**Melanoma** ***n* (%)**	
**Comprehensive care and information**																	
1. Up-to-date information		230 (34.0)	68 (23.4)			66 (25.8)		43 (41.7)		26 (25.0)			39 (40.2)			22 (27.8)	
2. Information for others		173 (25.6)	65 (22.3)			55 (21.5)		40 (38.8)		27 (26.0)			20 (20.6)			19 (24.1)	
3. Understandable information		296 (43.7))	102 (35.1)			103 (40.2)		60 (58.3)		44 (42.3)			46 (47.4)			34 (43.0)	
4. Best medical care		331 (48.9)	104 (35.7)			108 (42.2)		63 (61.2)		41 (39.4)			45 (46.4)			33 (41.8)	
5. Local healthcare service		344 (50.8)	119 (40.9)			133 (61.2)		63 (61.2)		48 (46.2)			48 (49.5)			32 (40.5)	
6. Manage health with team		393 (58.1)	149 (51.2)			152 (59.4)		79 (76.7)		54 (51.9)			55 (56.7)			41 (51.9)	
7. Doctors talk to each other		375 (55.4)	139 (47.8)			146 (57.0)		68 (66.0)		50 (48.1)			50 (51.5)			42 (53.2)	
8. Complaints addressed		389 (57.5)	148 (50.9)			166 (64.8)		72 (69.9)		57 (54.8)			48 (62.0)			51 (64.6)	
9. Complimentary therapy		350 (51.7)	72 (24.7)			97 (37.9)		65 (63.1)		38 (36.5)			41 (42.3)			22 (27.8)	
**Physical effects**																	
11. Manage side effects		232 (34.3)	39 (13.4)			36 (14.1)		41 (39.8)		24 (23.1)			34 (35.1)			6 (7.6)	
12. Changes to quality of life		208 (30.7)	21 (7.2)			39 (15.2)		43 (41.7)		17 (16.3)			30 (30.9)			7 (8.9)	
13. Fertility		33 (4.9)	23 (7.9)			14 (5.5)		20 (19.4)		1 (1.0)			9 (9.3)			2 (2.5)	
26. Changes to my body		137 (20.2)	8 (2.7)			28 (10.9)		29 (28.2)		13 (12.5)			20 (20.6)			1 (1.3)	
**Psychological effects**																	
10. Reduce stress in my life		239 (35.3)	29 (10.0)			44 (17.2)		51 (49.5)		12 (11.5)			33 (34.0)			15 (19.0)	
19. Concerns about cancer coming back		201 (29.7)	42 (14.4)			51 (19.9)		43 (41.7)		23 (22.1)			34 (35.1)			17 (21.5)	
20. Emotional support for me		227 (33.5)	29 (10.0)			44 (17.2)		42 (40.8)		17 (16.3)			33 (34.0)			14 (17.7)	
24. Talking to others		194 (28.7)	36 (12.4)			42 (18.4)		43 (41.7)		22 (21.2)			28 (28.9)			6 (7.6)	
29. Move on with my life		147 (21.7)	16 (5.5)			26 (10.2)		28 (27.2)		11 (10.6)			22 (22.7)			5 (6.3)	
30. Changes to beliefs		177 (26.1)	18 (6.2)			30 (11.7)		34 (33.0)		13 (12.5)			25 (25.8)			10 (12.7)	
31. Acknowledging the impact		136 (20.1)	20 (6.9)			29 (11.3)		32 (31.1)		13 (12.5)			25 (25.8)			6 (7.6)	
32. Survivor expectations		148 (21.9)	15 (5.2)			24 (9.4)		34 (33.0)		9 (8.7)			23 (23.7)			6 (7.6)	
33. Decisions about my life		167 (24.7)	20 (6.9)			28 (10.9)		27 (26.2)		8 (7.7)			22 (22.7)			6 (7.6)	
34. Spiritual beliefs		67 (9.9)	8 (2.7)			16 (6.3)		20 (19.4)		4 (3.8)			8 (8.2)			1 (1.3)	
35. Make my life count		134 (19.8)	20 (6.9)			24 (9.4)		21 (20.4)		8 (7.7)			17 (17.5)			4 (5.1)	
**Practical issues**																	
14. Employment		114 (16.8)	4 (1.4)			22 (8.6)		28 (27.2)		10 (9.6)			11 (11.3)			5 (6.3)	
15. Financial support		247 (36.5)	41 (14.1)			56 (21.9)		48 (46.6)		20 (19.2)			32 (33.0)			11 (13.9)	
16. Life/travel insurance		122 (18.0)	12 (4.1)			27 (10.5)		34 (33.0)		7 (6.7)			7 (7.2)			6 (7.6)	
17. Legal services		121 (17.9)	17 (5.8)			28 (10.9)		39 (37.9)		8 (7.7)			16 (16.5)			4 (5.1)	
18. Accessible hospital parking		95 (14.0)	25 (8.6)			32 (12.5)		27 (26.2)		17 (16.3)			12 (12.4)			10 (12.7)	
28. Ongoing case manager		231 (34.1)	46 (15.8)			55 (21.5)		46 (44.7)		24 (23.1)			28 (28.9)			10 (12.7)	
**Relationships**																	
21. Support partner/family		152 (22.5)	34 (11.7)			36 (14.1)		38 (36.9)		13 (12.5)			29 (29.9)			7 (8.9)	
22. Impact on my relationship		146 (21.6)	42 (14.4)			28 (10.9)		33 (32.0)		9 (8.7)			25 (25.8)			6 (7.6)	
23. New relationships		96 (14.2)	21 (7.2)			17 (6.6)		27 (26.2)		10 (9.6)			19 (19.6)			2 (2.5)	
25. Handle social/work situations		109 (16.1)	21 (7.2)			29 (11.3)		38 (36.9)		11 (10.6)			21 (21.6)			5 (8.3)	
27. Problems with sex life		158 (23.3)	64 (22.2)			35 (13.7)		36 (35.0)		13 (12.5)			21 (21.6)			4 (5.1)	

Note: S = Surgery, RT = Radiotherapy, CT = Chemotherapy.

## Data Availability

The raw data supporting the conclusions of this article will be made available by the authors on request.
